# Cardiac Chamber Quantification by Echocardiography in Adults With Sickle Cell Disease: Need Attention to Eccentric Hypertrophy

**DOI:** 10.7759/cureus.15592

**Published:** 2021-06-11

**Authors:** Mahmut B Koyuncu, Anil Tombak, Ozcan Orscelik, Tolga Koseci, Ali Turker, Hakan Basir, Aydan Akdeniz, Eyup N Tiftik

**Affiliations:** 1 Hematology, Adana City Research and Training Hospital, Adana, TUR; 2 Hematology, Mersin University, Faculty of Medicine, Mersin, TUR; 3 Cardiology, Mersin University, Faculty of Medicine, Mersin, TUR; 4 Medical Oncology, Adana City Research and Training Hospital, Adana, TUR; 5 Internal Medicine, Mersin University, Faculty of Medicine, Mersin, TUR

**Keywords:** sickle cell disease complications, sickle cell disease: scd, cardiac complications, left ventricular mass index, relative wall thickness

## Abstract

Introduction and aim

Sickle cell anemia (SCA) is the most common hemoglobinopathy worldwide, and cardiovascular diseases are the most common causes of death. In these patients, cardiac remodeling begins from childhood and leads to sickle cell cardiomyopathy in the following years. Concentric hypertrophy and eccentric hypertrophy are known to predict early cardiac events. This study aims to reveal the relationship between cardiac remodeling types and survival in patients with SCA and investigate the factors that may affect left ventricular mass.

Materials and methods

A total of 146 patients with SCA were included in the study, and the left ventricular mass index (LVMI) and relative wall thickness (RWT) of the patients were calculated according to echocardiographic measurements, and the patients were categorized into normal, concentric remodeling (CR), concentric hypertrophy (CH), and eccentric hypertrophy (EH) groups.

Results

The median age of the patients is 32 (18-72). In logistic regression analysis, hemoglobin S (HbS) and ferritin levels were independent predictors for LVMI (p = 0.01 and p < 0.001, respectively). It was observed that 56 (38.4%) of the patients had normal left ventricles, 24 (16.4%) had CR, 21 (14.4%) had CH, and 45 (30.8%) had EH. 31 (21.2%) of the patients died. When we look at the survival curves, there was a statistically significant difference between the four groups (log-rank p < 0.001). It was observed that patients with EH were the group with the lowest probability of survival.

Conclusion

Cardiac death is one of the most common causes of death in patients with SCA. Early detection of cardiac disorders and starting treatment may be important in reducing mortality in these patients.

## Introduction

Sickle cell anemia (SCA) is the most common hemoglobinopathy globally, and its incidence is increasing [1]. Thanks to improvements in healthcare services, the average life expectancy in these patients has increased, but it is still 20-30 years shorter than the normal population [2]. Cardiovascular causes are one of the important causes of death, and it has been shown in autopsy studies that the most common organ involvement in patients with SCA is the heart [3]. Studies have shown that patients with SCA have an increased probability of developing cardiomyopathy and heart failure later in the life [4]. Although the effects of SCA on the right heart due to pulmonary hypertension are well known, its impact on the left heart are also increasingly understood. Meta-analyses have shown that left ventricular diastolic dysfunction is also quite common in these patients [5].

With the renin-angiotensin-aldosterone system (RAAS) activation due to chronic anemia and peripheral vascular dilatation, cardiac remodeling is triggered, and dilatation begins to occur in the left ventricle. This dilatation further triggers compensatory eccentric cardiac hypertrophy [6]. Conditions such as increased iron load (due to chronic blood transfusion) and pulmonary hypertension associated with decreased nitric oxide also contribute to ventricular hypertrophy. Many studies have shown that diastolic dysfunction is an independent risk factor for premature deaths in patients with SCA [7,8]. Besides these known mechanisms, it was understood that several newly defined molecular mechanisms also contribute to cardiac remodeling [9].

Cardiac remodeling types are determined as concentric remodeling (CR), concentric hypertrophy (CH), and eccentric hypertrophy (EH) [10]. Among these, concentric hypertrophy and eccentric hypertrophy are more likely to progress to heart failure within ten years than other types [10]. Studies evaluating left ventricular geometries in patients with SCA are available in the literature, but few studies on survival are available. 

This study aims to investigate the factors affecting left ventricular mass index (LVMI) in adult patients with SCA and to reveal the relationship between left ventricular geometry and overall survival.

## Materials and methods

Study sample

One hundred forty-six patients with SCA aged 18 years and older and visited Mersin University Faculty of Medicine Hematology Department between January 2015 and January 2016 and underwent echocardiography for routine cardiac screening to evaluate the survival status of the patients for at least five years were included in the study. Patients with pulmonary hypertension or systemic hypertension, patients with left ventricular ejection fraction below 50%, patients with renal insufficiency or diabetes, and cases with valve pathology on echocardiography were excluded. Patients' age, hemogram parameters, hemoglobin electrophoresis results, sickle cell anemia subtype, hemolysis parameters, serum ferritin levels, and body mass indexes were recorded. While calculating the survival times, the date of echocardiography (that was done during routine cardiac screening) was used as the starting date. The dates of the death were obtained from both the hospital electronic records and the electronic central population database of the Ministry of Health.

Left ventricle geometric examinations

**Figure 1:**
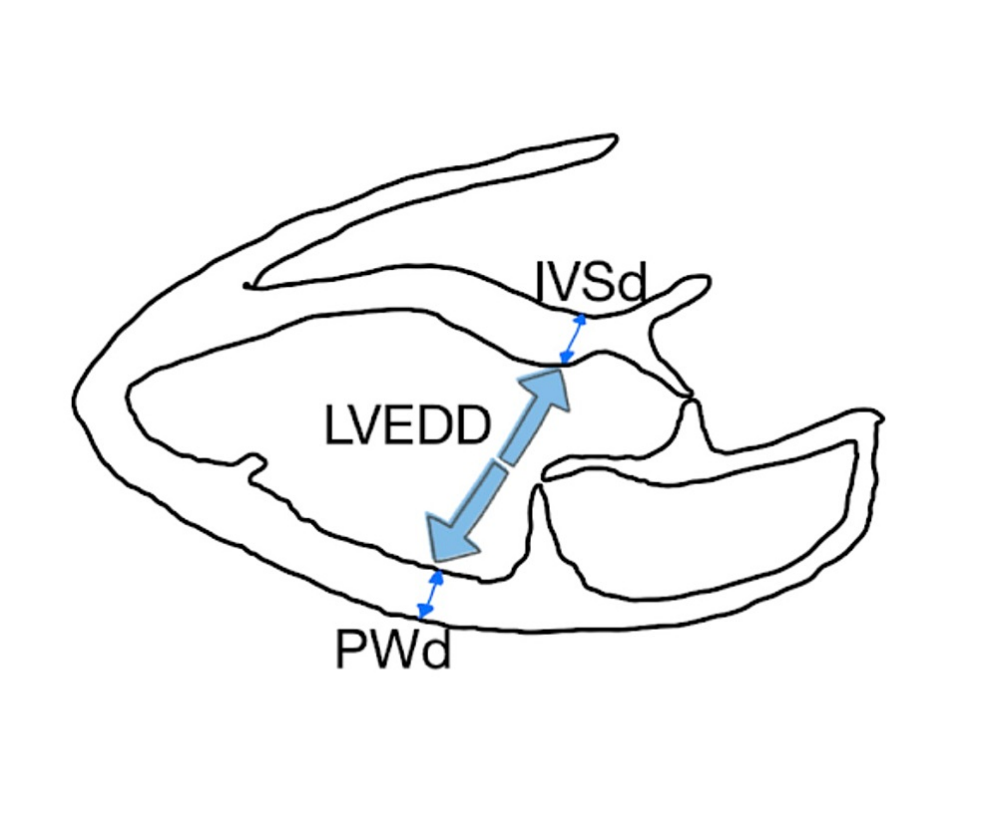
Left ventricular echocardiographic drawing and parameters used to calculate left ventricular mass index. IVSd: dimension of Intraventricular septum, LVEDD: left ventricle end-diastolic dimension, PWd: dimension of posterior wall.

Left ventricular mass (g) was calculated with Cube formula using left ventricular end-diastolic diameter (LVEDD), interventricular septum diameter (IVSd), and left ventricular posterior wall diameter (PWd) measurements (Figure 1).

**Figure 2:**
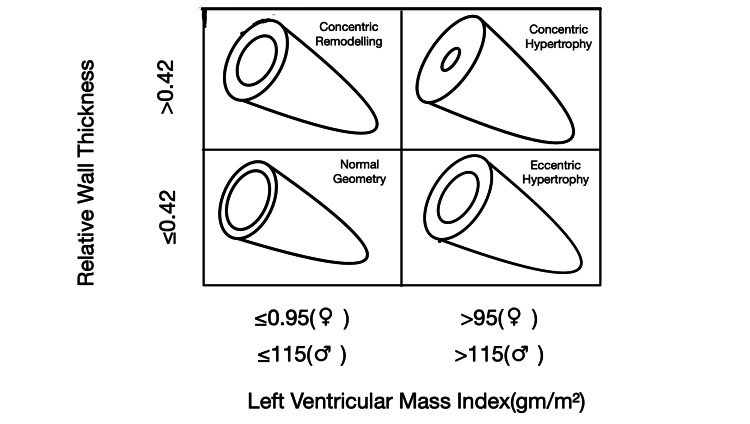
Determining the type of left ventricular geometry.

Left ventricular mass indexes (g/m^2^) were obtained by dividing the left ventricular masses by body mass index. Using the threshold values recommended by the American Echocardiography Society according to gender, the patients were grouped as patients with normal and increased left ventricular mass (115 g/m^2^ for men, 95 g/m^2^ for women). Relative wall thickness (RWT) was calculated using the formula RWT=2⋅PWd/LVEDD. The patients were grouped with a threshold value of 0.42 for RWT. Using RWT and LVMI values, the left ventricular geometry types of the patients were determined as shown in Figure 2 [11].

Statistical analysis

Data distribution characteristics were evaluated with the Kolmogorov-Smirnov test, skewness and kurtosis assessment, coefficients of variations, probability plots, and histogram. Since all continuous data in the study showed abnormal distribution, it was presented as the median (minimum-maximum). The correlation between biochemical measurements and LVMI was analyzed by the Spearman correlation test. After evaluating the parameters associated with the LVMI with univariate analysis, a multivariable logistic regression model was created with the appropriate parameters. The factors that could be independently related were determined. To determine the effects of impact factors on survival, the "Log-Rank" test, the test statistic in the Kaplan-Meier method, was used. Time to reach the relevant event (death) and time (month) was used as the variable containing the follow-up time. A value of p < 0.05 was considered statistically significant. In assessing the data, Statistical Package for the Social Sciences (SPSS) version 22 (IBM Corp., Armonk, NY) statistics package program has been used.

Ethical approval

This study was conducted in compliance with the principles of the Declaration of Helsinki. It was approved by the Mersin University Faculty of Medicine's Ethics Committee (Decision date: 03.03.2021 Number:2021/203).

## Results

Demographic, clinical, and echocardiographic measurement data of the patients are summarized in Table 1.

**Table 1:** Clinical, demographic and echocardiographic characteristics of the patients. EF: ejection fraction; HbS: hemoglobin S.

Variables	Patients (N = 146)
Age, years, median (min-max)	32 (18-72)
Gender	n (%)
Female	69 (47.2)
Male	77 (52.8)
Sickle cell subtype	n (%)
Hb SS	76 (52.1)
Hb Sbeta	70 (47.9)
Hemoglobin (g/dL), median (min-max)	8.65 (4.8-10.6)
Ferritin (ng/ml), median (min-max)	739 (38-2961)
HbS, %, median (min-max)	78.9 (47.1-94.2)
HbF, %, median (min-max)	7.7 (1-30)
Left ventricle EF, %, median (min-max)	63 (59/66)
Left ventricle mass (g), median (min-max)	176 (78-532)
Left ventricle mass index (g/m^2^), median (min-max)	101.5 (45-293)
Relative wall thickness, median (min-max)	0.38 (0.24-0.56)
Death, n (%)	31 (21.2)
Cardiac geometry	
Normal, n (%)	56 (38.4)
Concentric remodelling, n (%)	24 (16.4)
Concentric hypertrophy, n (%)	21 (14.4)
Eccentric hypertrophy, n (%)	45 (30.8)

Low-medium levels of correlations were detected between LVMI and age (r = 0.359, p < 0.001), HbS level (r = 0.201, p = 0.15), hemoglobin level (r = -0.293, p < 0.001), ferritin level (r = 0.546, p < 0.001) and HbF level (r = -0.266, p = 0.001). In the multivariate logistic regression model, HbS level and ferritin levels were determined as independent predictors (Table 2).

**Table 2:** Independent predictors for eccentric hypertrophy. B: coefficients; SE: standard error; OR: odds ratio; CI: confidence interval.

						95% CI for OR	
	B	SE	Wald	p-value	OR	Lower	Upper
HbS (%)	0.067	0.026	6.67	0.01	1.06	1.016	1.125
Ferritin (ng/ml)	0.002	0.00	17.97	<0.001	1.002	1.001	1.003

**Figure 3:**
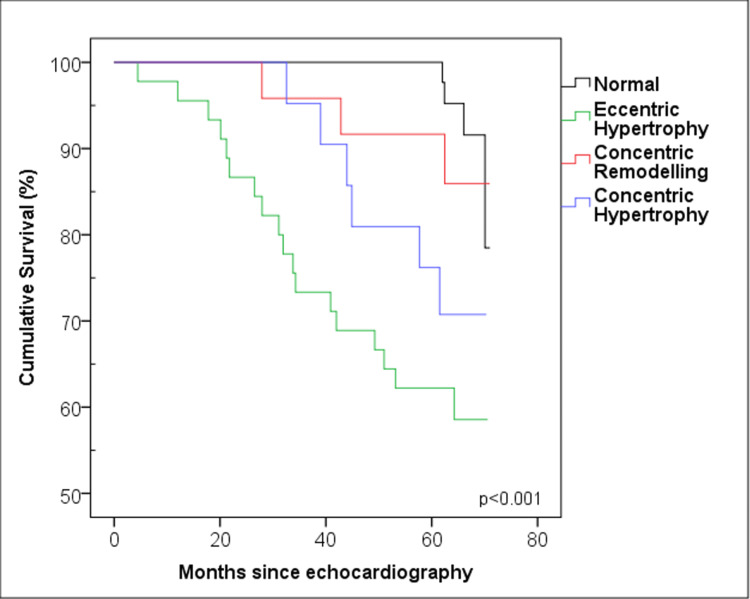
Survival curves for four types of cardiac geometry.

It was observed that 31 (21.2%) of the patients died during the follow-up. When the left ventricular geometric properties of the patients were examined in four categories (normal, CR, CH, and EH), a significant difference in survival was found between the groups (log-rank p < 0.001) (Figure 3).

**Figure 4:**
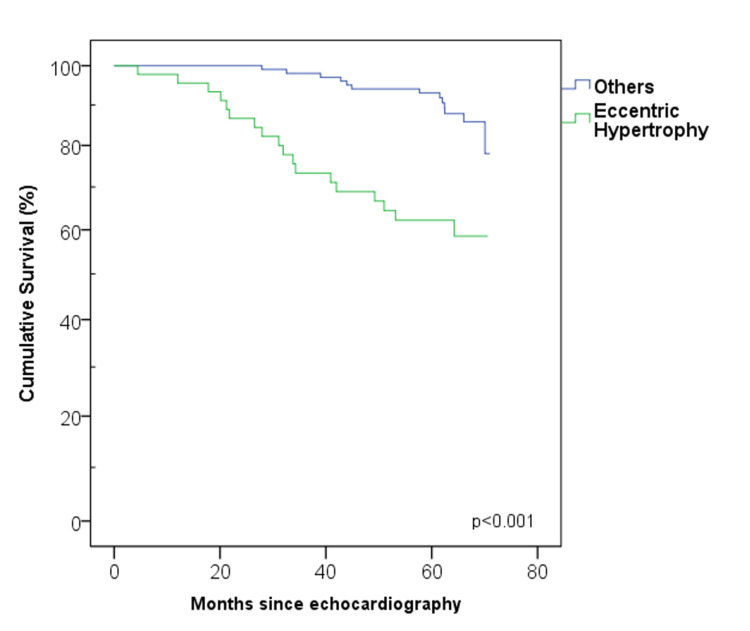
Survival curves obtained when cardiac geometries are categorized into two subgroups (eccentric hypertrophy and others).

Median survival time was not reached for any group during the follow-up period. When the left ventricular geometry features were examined in two categories (EH and others), a significant statistical difference was observed (log-rank p < 0.001) (Figure 4).

When the life tables of the groups were assessed, the 60th-month survival rates were found to be 96% in patients with normal geometry, 86% in patients with CR, 76% in patients with CD, and 62% in patients with EH.

## Discussion

This study is a retrospective cohort study aiming to see the effect of cardiac geometry types on survival probability in patients with SCA. This study is critical because the number of studies evaluating cardiac geometries in adult patients with SCA are very few. We could not find a study evaluating survival according to cardiac geometry in the literature. It is also important that the follow-up period of the patients in the study should be at least five years. Our study has shown that eccentric hypertrophy is common in adult patients with SCA and is also the geometry type in which all-cause death is most common.

Calculating left ventricular mass from left ventricular measurements obtained by 2D echocardiography using the linear method (the Cube formula) is a widely used and is a rapid method. Its prognostic importance has been established by many studies. It can be applied quickly and is a suitable method for screening large populations. The disadvantages of this method are its ability to overestimate the left ventricular mass by up to 20%, and the accuracy of the measurement is significantly affected by minor measurement differences [11]. Studies using this method have shown that geometry types are important indicators in predicting cardiovascular events [12]. In mortality studies, it was reported that patients with concentric hypertrophy and eccentric hypertrophy have higher mortality than other types [13].

Although many studies in the literature evaluate left ventricular masses in patients with sickle cell anemia, especially in the pediatric patient group, there is no existing study, so far, on overall survival [14]. The number of such studies are much lower in adult patients with SCA. Damy et al. showed that dilatation in the left chambers of the heart is quite common in these patients and is associated with the severity of anemia and hemolysis [15]. In the study of Vasconcelos et al., it was shown that LVMI and cardiac dilatation findings increased significantly in patients with SCA compared to the normal population. However, left ventricular functions were preserved, as in our study. In the same study, it was found that serum ferritin levels correlated with left ventricular mass as seen in our study, and systemic blood pressure, ferritin levels, and tricuspid regurgitation (TR) velocity were independent predictors in the logistic regression model [16]. Since patients with hypertension and high TR velocity are excluded in our patient population, it can be considered a more accurate model for evaluating the factors affecting LVMI in asymptomatic patients with SCA. In studies conducted with patients in the pediatric age group, a relationship between serum ferritin and left ventricular mass has not been shown. It can be concluded that high ferritin levels probably cause an increase in left ventricular mass over the years [17]. In the study of Yang et al., iron chelation therapy was shown to prevent iron-induced cardiomyopathies in mouse models, but human studies on this subject are not yet sufficient [18]. In our study, serum ferritin level is an independent risk factor for cardiac hypertrophy, and effective chelation therapy may benefit these patients. However, several studies have shown that myocardial iron overload is not common in patients with SCA [6]. Therefore, other non-ferrous mechanisms are more responsible for the cardiac geometry changes seen in these patients. In these patients, the reason for the excessive accumulation of iron in the heart is that iron becomes more prone to storage in the reticuloendothelial system due to chronic inflammation associated with the disease [19].

The chronic inflammation and microvascular occlusion observed in these patients predisposes to the formation of myocardial fibrosis. It was shown that myocardial fibrosis is essentially related to diastolic dysfunction and restrictive cardiomyopathy in these patients [7]. Many studies have shown that IL-18 and certain genetic factors play a role in the development of myocardial fibrosis and sickle cell cardiomyopathy [20, 21]. In the future, it is expected that treatments targeting IL-18 will be developed in patients with sickle cell cardiac geometry disorders. Angiotensin-converting enzyme (ACE) inhibitors, Angiotensin receptor blockers, and calcium channel blockers are known to slow the cardiac remodeling and prevent cardiac fibrosis. Although the cardiac remodeling mechanisms in patients with sickle cell anemia are pretty diverse, the same treatments are used to avoid remodeling in these patients [22]. In one study, L-carnitine therapy was shown to improve diastolic functions in children with SCA [23]. The search for new therapies for cardiac remodeling is underway, with many ongoing clinical trials.

Regarding the limitations of the study, first of all, this study is a retrospective cohort study. We believe that prospective studies are needed on this subject. Since the exact causes of death of the patients are unknown, mortality analyses were made based on mortality due to all causes. It would be better if we could analyze deaths from cardiovascular causes. One of the limitations of the study is that the echocardiography of the patients was not performed by the same cardiologist. Besides, analysis of diastolic functions could not be made since diastolic measurements were not made in every patient.

## Conclusions

In our study, it was shown that 45.2% of the patients have concentric or eccentric cardiac hypertrophy. When it is considered that this rate is 14% even in the population with heart failure, it is better understood how frequently it is seen. This study shows that cardiac geometry changes are prevalent and overlooked in patients with SCA. It is vital to recognize cardiac remodeling at an early stage and initiate preventive interventions in these patients. Prospective studies are needed to examine the positive contribution of current therapies on mortality, especially in patients with CH and EH of the cardiac muscle.
